# Lobbying in the Sunlight: A Scoping Review of Frameworks to Measure the Accessibility of Lobbying Disclosures

**DOI:** 10.34172/ijhpm.8497

**Published:** 2024-10-08

**Authors:** Jennifer Lacy-Nichols, Hedeeyeh Baradar, Eric Crosbie, Katherine Cullerton

**Affiliations:** ^1^Centre for Health Policy, Melbourne School of Population and Global Health, The University of Melbourne, Melbourne, VIC, Australia.; ^2^School of Public Health, University of Nevada Reno, Reno, NV, USA.; ^3^School of Public Health, The University of Queensland, Brisbane, QLD, Australia.

**Keywords:** Lobbying, Transparency, Framework, Corporate Political Activity, Commercial Determinants

## Abstract

**Background:**

While anyone can lobby governments, most lobbying is driven by commercial interests. Due to limited government disclosures, it is often challenging to get a clear picture of who is lobbying whom or why. To help make lobbying more visible to the public, we set out to develop a framework of key criteria for best practice government lobbying disclosures.

**Methods:**

We undertook a systematic scoping review of peer-reviewed and grey literature to identify frameworks for measuring or evaluating lobbying transparency. We screened the titles and abstracts of 1727 peer-reviewed and 184 grey literature articles, assessing 230 articles for eligibility. Following screening, we included 15 frameworks from six peer-reviewed and nine grey literature articles in our review. To create our framework of lobbying disclosures, we thematically coded the 15 included frameworks and used an iterative process to synthesise categories.

**Results:**

The 15 frameworks covered more than only lobbying disclosures, with the most common other theme about enforcement and compliance. Most frameworks were developed to evaluate lobbying transparency in particular jurisdictions, with the United States the most common. Of the 15 frameworks analysed, those developed by non-governmental organizations (NGOs) focused mainly on improving lobbying regulations, while most peer-reviewed studies developed frameworks to measure, compare and evaluate lobbying regulations. We developed a Framework fOr Comprehensive and Accessible Lobbying (FOCAL). It comprised eight primary categories (scope, timeliness, openness, descriptors, revolving door, relationships, financials, and contact log) covering 50 total indicators.

**Conclusion:**

Government transparency plays a crucial role in facilitating access to information about commercial political activities like lobbying. Our framework (FOCAL) offers a template for policy-makers to develop or strengthen regulations to improve lobbying transparency so commercial political influence strategies are more visible and subject to public scrutiny. This is an important step towards rebalancing influence toward the public interest.

## Introduction

 The capacity to engage with and influence governments is heavily weighted towards commercial interests. This engagement takes different forms, including political donations, meeting with public servants, public tenders, consulting, participation in policy development, grassroots advocacy campaigns, funding “astroturf” organisations to camouflage political activities and hiring former politicians and public servants (a practice often referred to as the revolving door).^1-3^ A large body of scholarship and investigative reporting has documented extensive examples of commercial actors blocking, weakening and delaying public policies, especially in public health.^4-7^ Prominent examples include the tobacco industry’s use of front groups to attack and divide tobacco control allies and the firearm industry’s opposition to efforts in the United States to strengthen gun control.^8,9^

 Engagement with government is not inherently problematic. Civic engagement and participation is essential to a healthy democracy.^10^ Many individuals, advocacy groups, charities, think tanks, not-for-profits, industry associations and for-profit businesses engage with governments. Nonetheless, empirical studies have shown that business interests consistently dominate lobbying and political donation activities.^11^ This raises substantive concerns about government decision-making being biased towards vested commercial interests.^12^ Of course, this is not always the case. Indeed, the history of tobacco control highlights the successful champion of public health over commercial interests. Nonetheless, lobbying and other political activities are often hidden from the public. This makes it difficult to understand who is trying to influence government decision-making and why. This risks a loss of public trust in governments as well as governments making decisions not in the public interest. To understand the degree to which governments prioritise commercial over public interests, we must first be able to measure the extent and nature of commercial political influence. In practice, information about commercial political activities – lobbying especially – is frequently lacking. This is especially concerning in low-income contexts, where commercial actors often use more aggressive strategies to oppose public health policies.^13,14^ Previous studies analysing lobbying have documented the challenges of doing so – many of which arise from inadequate disclosures and poorly designed platforms and databases to share lobbying information.^15,16^ Other research suggests that as business reputations become more negative, they engage in political strategies that are less visible and more controversial.^17^ In our own research, we have similarly faced challenges accessing, extracting, cleaning, coding and analysing lobbying data.^18^

 Government datasets are an important source of information about the political activities undertaken by businesses, industry associations and professional lobbyists, as well as non-governmental organizations (NGOs) and other interest groups. These datasets can take many forms, including lobbyist registers, open diaries/agendas, political donation reports, conflict of interest disclosures, public repositories of policy submissions and records of committee hearings. However, these datasets are not routinely available in many jurisdictions around the world. A 2021 report from the Global Data Barometer found that only 19 of 109 surveyed countries had a lobbyist register available online.^19^ Many of these are high-income countries, highlighting the further challenge low- and middle-income countries (LMICs) face in addressing commercial political influence.

 Not only are data sources about political activities often missing, when present, they do not provide information that is sufficiently complete, timely or easily searchable. This points to the difference between making information merely available and making it truly accessible—the latter aligns more with Open Data principles—data must be credible, complete, timely, comprehensible, and comparable.^20^ A report from Transparency International highlighted limitations with the current data made available in the European Union (EU) concerning lobbying, finding that data openness for lobbyist meetings was poor, with only “average” data quality (eg, information located across 98 different websites, not machine readable).^21^

 There have been several studies analysing the transparency and robustness of lobbying regulation. Some of these studies have developed benchmarking indices and frameworks to assess lobbying regulations. Chari and colleagues^22^ have done extensive work comparing different indices, concluding that the “Hired Guns” methodology developed by the US-based Center for Public Integrity had the best validity and replicability. Other research teams have developed their own set of criteria to assess lobbying transparency, with Laboutková and Vymětal^20^ creating perhaps the most extensive model, with 158 indicators covering four domains: lobbying, targets of lobbying, sunshine principles and monitoring and sanctioning systems. These studies often go far beyond lobbyist registers to examine what makes for a “transparent lobbying environment” – while this includes disclosure of lobbying activities, it also includes broader transparency measures around government decision-making such as the publishing of legislative footprints or ministerial diaries.^20^ Here we examine the narrower topic of lobbying disclosures, ie, how information is shared in the public domain.

 Our approach focused on the two most common forms of lobbying disclosures: lobbyist registers and open agendas. Lobbyist registers can take many forms, but many tend to provide three pieces of information: (1) individuals or organisations engaging in lobbying, (2) government representative(s) being targeted, and (3) communication (such as the date of the meeting and topic discussed). Open agendas are essentially a record of a public servant’s or politician’s meetings, often including the date, time, location, attendees, and topics discussed. These two data sources complement one another, and often provide overlapping information.

 We note that transparent lobbying encompasses more than just disclosures, including many of the elements detailed by Laboutková and Vymětal^20^ such as codes of conduct, conflicts of interests, sunshine principles (disclosures about law- and decision-making processes), legislative footprint and freedom of information. Indeed, several of the above indices emphasise the importance of assessing compliance mechanisms, as some lobbying regulations may have stringent disclosure requirements, yet lack adequate enforcement mechanisms, especially in LMICs. While these other elements of transparency are outside the scope of this study, we direct interested readers to other studies on this topic.^20,22^

 The impetus for this project came from our practical experience of trying to monitor commercial political activities, and the challenges and frustrations we and others internationally faced in accessing data about lobbying. Our aim was to develop a framework of what information could be made public in government disclosures about lobbying to ensure a comprehensive approach to lobbying disclosures. To do this, conducted a scoping review to identify what frameworks have been developed to measure lobbying disclosure. We then synthesised these frameworks to develop a comprehensive framework of key criteria and indicators to evaluate government lobbying disclosures. We note that this framework may not be applicable in the same way across all political systems, and that alterations may be necessary to account for the different systems and rules in place. However, it presents a potential baseline of relevant information that governments could make public about lobbying.

 We hope that this approach and our framework offer a useful step forward in efforts to increase the transparency and accessibility of information on commercial political activity. Robust lobbying disclosure regulations are useful for people (like us) who study lobbying. They are also important for society, as increased transparency can foster citizen engagement, which in turn can strengthen democracy.^23^ In our discussion, we reflect on opportunities to apply this framework to other political practices, such as donations and the revolving door.

## Methods

 We conducted a systematic scoping review to identify frameworks for measuring lobbying disclosure. We thematically grouped the indicators identified in the frameworks to develop a Framework fOr Comprehensive and Accessible Lobbying (FOCAL). We present this framework in the results and discuss possible applications in the discussion. Our scoping review followed the five step approach set out by Arksey and O’Malley^24^: (1) identifying the research question; (2) identifying relevant literature; (3) screening the literature; (4) “charting” the data; and (5) summarising and reporting the results. Our scoping review seeks to explore the following question: what frameworks have been developed to measure lobbying disclosure?

###  Search Strategies

 For our review, we were interested in identifying novel frameworks that had been created to measure or evaluate lobbying disclosures. With this focus, in February 2023 the authors developed a set of search terms comprising three conceptual categories: framework, lobbying and disclosure. In our initial searches, we found that many studies and organisations used the term transparency to refer to disclosures, so this term was used for our initial searches. In March 2023, JLN completed searches for these terms across five databases: Scopus, Web of Science, ProQuest, JSTOR, and Business Source Complete. Searches were tailored to meet database formatting requirements and limited to titles, abstracts and key words, as broader searches yielded irrelevant results. Our search strategy for Scopus was: (TITLE-ABS-KEY (framework* OR model* OR principle* OR schem* OR criteri* OR indicator* OR indice* OR index OR assessment* OR evaluation* OR structure*) AND TITLE-ABS-KEY (lobby*) AND TITLE-ABS-KEY (transparen* OR disclos* OR register* OR registr* OR log OR agenda* OR diar* OR contact*) OR ALL (“lobbyist code” OR “Lobbying code” OR “contact log” OR “open agenda” OR “open diary” OR “lobbying regulation” OR “Lobbyist regulation”)). JLN searched the databases on March 8, 2023, downloaded all records (n = 2535) and imported into Endnote X9 where duplicates were removed.

**Figure 1:**
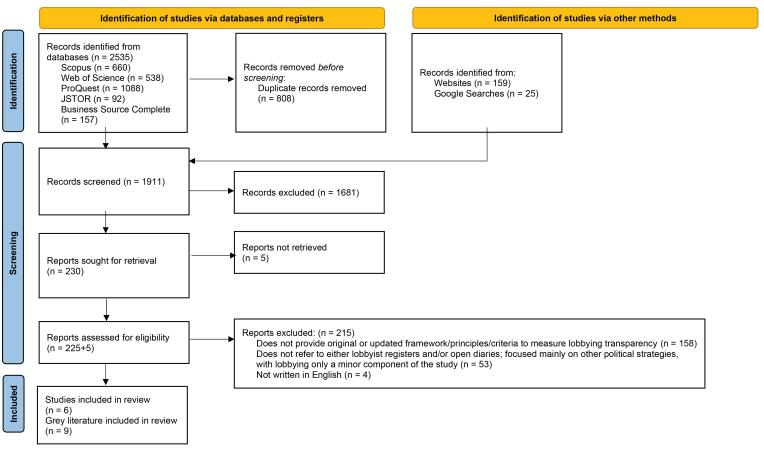
PRISMA Flow Diagram. Abbreviation: PRISMA, Preferred Reporting Items for Systematic Reviews and Meta-Analyses.

 Between February and April 2023, we also searched the grey literature for relevant frameworks, as many NGOs play a prominent role in monitoring lobbying and advocating for increased transparency. We used the approach developed by Godin et al^25^ to systematically analyse the grey literature. We conducted two searches with Google’s Advanced Search feature using similar terms to the database searches. We also searched the websites of 23 organisations with expertise on lobbying and transparency. This list was created based on the knowledge of the authors and building on similar studies.^26,27^ Each website was searched for the terms transparency and lobby (as other terms did not yield relevant results). 184 documents were downloaded for screening. We document all database and grey literature search strategies in Supplementary file 1 (See also Figure 1).

###  Screening and Data Extraction

 Between April and May 2023, HB screened the titles and abstracts/summaries of 1911 peer-reviewed and grey literature records, with JLN double screening 10 percent. After screening, 225 documents were assessed for eligibility. In addition, five other documents were identified through backward searches. During our review of the full text of these 230 documents, we identified many documents that provided specific recommendations to improve lobbying transparency but did not develop structured frameworks or indices that set out what should be included in lobbying disclosures. To ensure a feasible scope of analysis, we excluded these more ad hoc recommendations to improve lobbying transparency, and limited our included studies to those that provided specific frameworks or sets of detailed criteria for lobbying disclosures (See Table 1 for our inclusion criteria). We note that our study was limited to frameworks addressing two forms of lobbying disclosure: lobbyist registers and open agendas (See introduction for definitions). While transparent lobbying includes other elements (such as whistleblower protections or enforcement mechanisms), for feasibility we limited our study to the practice of disclosure. We included 15 reports and studies in our final sample.

**Table 1:** Inclusion and Exclusion Criteria

**Inclusion**	**Exclusion**
English language	Not written in English
Must focus on lobbyist registers and/or open diaries (though can mention other forms of political activity, such as donations)	Does not refer to either lobbyist registers and/or open diaries; focused mainly on other political strategies, with lobbying only a minor component of the study
Provides original or updated framework/structured list/specific criteria of elements that should be disclosed/made transparent about lobbying Notes: Does not need to provide quantitative indicators or thresholds for evaluation; specific criteria are sufficient (eg, “provide the purpose of the lobbying communication,” “data is interoperable”) Does not need to apply framework; can be conceptual Can analyse/evaluate/compare a country's lobbying transparency/regulation but would need to use a novel framework to do so The framework does not need to be globally applicable – could be used for only one context	Does not provide original or updated framework/structured list/specific criteria of elements that should be disclosed/made transparent about lobbying Instead, may: Only list high-level principles (eg, “lobbyists should disclose activities”) with no specific criteria for disclosure elements Analyse importance/impact lobbying transparency (but not provide framework to measure/benchmark this) Apply a previously developed framework (in which case, we sourced original framework) Analyse evolution of framework/ principles Analyse process of implementing framework/ principles, including facilitators/impediments Descriptions of actual registers and diaries - the content of these will be analysed in second phase of study Provides recommendations for lobbyists and/or companies to lobby responsibly, but not for governments to act on

 We extracted data on the characteristics of each report, including: title, year, authors, research question, methods, country focus, policy/register focus, whether the framework was conceptual or applied, the number and title of categories in the framework, whether indicators were weighted and the total number of indicators in the framework. Table 2 in our results provides a summary of the studies.

**Table 2:** Summary of Data Charting

**Author/ Organisation**	**Year**	**Research Question/Aim**	**Name of Framework**	**Methods Explained**	**Where Applied?**	**How Many Categories?**	**List All Category Titles**	**Total Number of Items in Framework**
Opheim C.	1991	To examines what factors account for the stringency of a state's lobby regulation laws and enforcement procedures	Index of state lobbying regulation law	Partly	US states	3	Statutory definition of a lobbyist; frequency and quality of disclosure; oversight and enforcement of regulations	22
Newmark A.	2005	To construct a replicable measure of lobbying regulation and analyse how lobbying regulation has changed	Index to measuring state lobbying regulation	Partly	US states	3	Definitions; frequency of reporting requirements; prohibited activities; disclosure requirements	18
Center for Public Integrity^a^	2007	To be able to rank states against the quality of their lobbying disclosure requirements	Hired Guns	Partly	US states	8	Definition of lobbyist; individual registration; individual spending disclosure; employer spending disclosure; electronic filing; public access; enforcement; revolving door provision	48
Pacific Research Institute^a^	2010	Improve understanding of taxpayer-funded lobbying (ie, government and quasi-government organizations) and assess disclosure and access to information across US states	State disclosure law criteria	Yes	US states	State disclosure law criteria (5) State lobbying information accessibility criteria (8)	*State disclosure law criteria: * Registration requirements; exemptions for government; defining public entities; materiality; disclosure *State lobbying information accessibility criteria: * Data availability; website existence; website identification; current data availability; historical data availability; data format; sorting data; simultaneous sorting	State disclosure law criteria (47) State lobbying information accessibility criteria (22)
Holman C; Luneburg W.	2012	"To discern best practices for achieving transparency through lobbying regulation" and to "offer recommendations on how to enhance transparency in policy-making"	Elements of lobbying regime	No	United States, Canada, France, Georgia, Germany, Lithuania, Macedonia, Poland, European Parliament, European Commission, Austria, Slovenia	US/Canada (7) Europe (8)	*US/Canada: * Specifies the type of activity that attracts a registration obligation; Entities that must register; disclosure of basic information about the registrant (for example, name, address, client) and the expected areas/issues of to be lobbied plus; Periodic reports required of lobbying and related activity covering; Administration of disclosure regime; Internet-accessible and searchable databases of information; A lobbyist code of conduct *Europe: * Mandatory or voluntary registration; access pass to lawmakers; lobbyist registrants; covered officials; registrants disclose; fines/imprisonment for violations; internet access to lobbying records; code of conduct required for registered lobbyists	US/Canada (25)Europe (21)
ALTER-EU^a^	2013	Provide list of reforms required to improve disclosure requirements of the EU Transparency Register.	Lobby disclosure requirements	No	EU Transparency Register	11	Financial disclosure requirements; transparency on funding sources; names of lobbyists and revolving door listings; issues lobbied on; securing up-to-date information; lobby firms’ clients; obliging registrants to disclose lobby consultancies and law firms assisting their lobbying; tackling the problem of under-reporting the number of lobbyists; more comprehensive and effective data checking; better public scrutiny; pro-active transparency	Descriptive text
Access Info Europe, Open Knowledge, Sunlight Foundation, Transparency International^a^	2015	"The Standards aim at providing clear guidance to policy-makers, governments and international organisations that are thinking of or are in the process of enacting lobbying legislation. They also serve as a reference point for civil society organisations to campaign in their countries to ensure that efforts to regulate lobbying are robust, comprehensive and effective"	International Standards for Lobbying Regulation	Partly	[Not applied]	7	Guiding principles; regulatory scope; transparency; integrity; participation & access; oversight, management and sanctions; regulatory framework design	72 (including 34 granular points)
Centre for Research on Multinational Corporations^a^	2016	Assess the Ministry of Finance and banks in the Netherlands for transparency, openness to citizen input, equality of access, balance and public interest, accountability	Framework was unnamed	Yes	Netherlands	12	Legally binding regulations; be transparent in order to protect the right to know; protect the right to be heard; protect the integrity of the democratic legislative decision-making process; ensure that the public interest is weighed fairly against all other interests and information; exercise more accountability about lobbying activities; a comprehensive transparency policy; better access for citizens, civil society organisations and diverse stakeholders to give input to the legislative processes; ensure all interests are weighed seriously; public information is to be improved and enhanced about lobbying activities undertaken and the positions held by the bank on financial legislative proposals; ensure integrity of the banks’ interactions with, and lobbying of, legislative authorities; develop a comprehensive policy on interaction and lobbying on legislative proposals	Descriptive text
Council of Europe^a^	2017	Develop recommendations for governments of EU member states to promote and increase transparency of lobbying activities	Guiding principles on devising policy at national level to regulate lobbying	Yes	[Not applied]	11	Definitions; objective of legal regulation; activities subject to legal regulation; freedom of expression, political activities and participation in public life; transparency; public registers of lobbyists; standards of ethical behaviour for lobbyists; sanctions; public sector integrity; oversight, advice and awareness; review	42
Newmark A.	2017	"First, how have political scientists and various organizations examined lobbying regulations in recent years? Second, how can we construct a valid measure of lobbying regulation? Third, how have these laws changed over the past decade?"	2015 Measure of lobbying regulation	Yes	US states	3	Definition; prohibited activities; disclosure	19
Carnstone Partners Ltd; Meridian Institute^a^	2020	To provide guidance on what responsible lobbying should look like for companies/civil society etc	The Responsible Lobbying Framework	Partly	[Not applied]	6	Definition; general disclosure requirements; financial disclosure requirements; timeliness, quality, and accessibility; integrity and ethics; enforcement and compliance	23
Roth A.S.	2020	To develop a tool to assess the robustness of lobbying regulations	Lobbying regulation robustness index	Yes	Austria, Australia, Canada, the EU, France, Germany, Lithuania, Mexico, the Netherlands, Poland, Slovenia, the UK, and the US	6	Definition; General disclosure requirements; financial disclosure requirements; timeliness, quality & accessibility; integrity & ethics; enforcement & compliance	23
Bednárová P.	2020	"To evaluate the lobbying regulation system in the draft Lobbying Act in the Czech Republic and to compare it with regulation models in selected European countries"	CII/HG methodology	Partly	Czech Republic; Austria; Poland; Slovenia; Hungary; Slovakia	8 (CII, HG)	Definition of lobbyists; individual registration; individual spending disclosure; employer spending disclosure; electronic filling; public access; enforcement; revolving door provision	17 included from HG + 19 included from CII = 36 total
Independent Broad-based Anti-corruption Commission^a^	2022	To present options for reforming Victorian legislation around lobbying and donations	Recommendation 3	Yes	[Not applied]	8	Defines the following in legislation; ensures members of parliament who initiate meetings with a minister or their adviser; requires that lobbyists document their contacts with government representatives, and that this information is published via an easily accessible and searchable register; mandates the publication of extracts or summaries of ministerial diaries and ministerial staff diaries on a monthly basis, capturing any form of meeting or event (such as attendance at fundraisers); ensures that interactions between a lobbyist and a minister or their staff are transparent; ensures that interactions between lobbyists and electorate officers are transparent; prohibits success fees; ensures that a lobbyist cannot lobby an elected official whose election they have supported directly or indirectly, for example, through donations or in-kind support to a campaign	23
Laboutková Š.; Vymatal P.	2022	"What are the determinants of transparent lobbying that is associated with the decision-making process? How do the relevant measures related to lobbying transparency contribute to [institutional quality] evaluation?"	Catalogue of transparent lobbying environments	Yes	[Not applied]	16 (grouped under 4 sections)	Lobbyists (register; codes of conduct; disclosure of activities)Targets of lobbying (Codes of Conduct; revolving doors; conflicts of interest; Disclosures of politicians/senior public employees) Sunshine principles (Rules on legislative process; rules on decision-making; rules on consultations; legislative footprint; Open Government data; political parties funding; freedom of information) Monitoring and sanctioning system (oversight; sanctions)	158

Abbreviations: EU, European Union; ALTER-EU, Alliance for Lobbying Transparency and Ethics Regulation; CII, Cost-Indicator Index; HG, Hired Guns. ^a^Grey literature.

###  Creation of a Lobbying Disclosure Framework

 To create a framework to assess the quality of lobbying disclosures, we thematically coded the 15 frameworks included in our final set. Based on an initial review of the 15 frameworks and the literature in our scoping review, we developed a preliminary list of coding categories. Building on the approach used in the Global Data Barometer, we focused on indicators measuring *what* information is disclosed in registers, and *how* information is disclosed. We excluded the following indicators/aspects of transparency as out of scope for this project: enforcement/compliance, sanctions, ethics/integrity laws, cooling off period requirements, how the public accesses the policy process.

**Figure 2:**
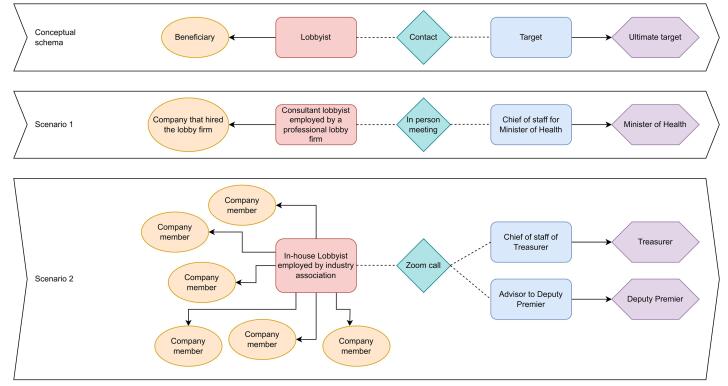
The Dynamics of Lobbying – a Conceptual Schema.

 To guide our coding and analysis of the frameworks, we created a conceptual schema of the dynamics of lobbying to distinguish between the various actors and interests involved (Figure 2). This helped guide our consideration of how information about these different aspects of lobbying could be disclosed.

 Between June and August 2023, we used QSR NVivo to code the frameworks, coding a total of 248 items. We took an iterative approach to modifying our coding framework as new categories emerged. We were primarily interested in indicators that could be assessed by viewing a register (eg, place of meeting is disclosed; names of all attendees are disclosed). However, two categories could only be assessed by reviewing legislation: (1) definitions of lobbyists, lobbying targets and lobbying activities; and (2) requirements about the frequency of disclosures. The categories and organisation of our framework was discussed and revised until consensus was reached. When decisions were made about consolidating different disclosure requirements from the 15 frameworks, we preferenced the most rigorous indicators. All authors reviewed and collaborated on defining and organising these categories.

## Results

###  Lobbying Transparency Frameworks

 Between 1991 and 2022, 15 frameworks were published with a focus on lobbying transparency and disclosure. Six were from the peer-reviewed literature, six from NGOs, two from government, and one from a master’s research project (supervised by the lobbying regulation expert Chari). With the exception of the “Hired Guns” methodology from the Center for Public Integrity, the NGO reports developed recommendations to improve lobbying regulations. In contrast, most peer-reviewed studies developed frameworks to measure and benchmark lobbying regulations. Some of the peer-reviewed studies had additional aims, including to analyse changes in regulations over time,^28,29^ to explain why some lobbying regimes are stricter or weaker than others,^30^ to evaluate and compare lobbying regulations^31^ and to identify best practice in lobbying regulation.^32^ The frameworks from the Center for Public Integrity,^33^ Roth,^34^ and Laboutková & Vymětal^20^ presented tools to evaluate the quality of lobbying regulations.

 Several of the frameworks were developed to focus on specific jurisdictions. Five studies focused on state-level lobbying regulations in the United States,^28-30,33,35^ two developed recommendations for the EU Transparency Register,^36,37^ and one focused on the Netherlands.^38^ Three studies compared countries in Europe and North America.^31,32,34^ Both government reports developed recommendations for specific registers (the EU Transparency register and the Victorian lobbyist register in Australia).^37,39^ The remaining three frameworks were conceptual only and designed to apply to lobbying disclosures in general.^20,40,41^

 Seven papers discussed how they created their frameworks in detail (noting that the 2022 Laboutková and Vymětal paper was a synthesis paper with its methods discussed in earlier studies).^20,29,34,35,37-39^ Six papers provided very brief descriptions of their methods,^28,30,31,33,40,41^ while two papers did not provide explanations for how their frameworks were created.^32,36^ Several of the studies built on the earlier frameworks, in particular Opheim’s 1991 index and the Center for Public Integrity’s 2007 Hired Guns methodology.

 Of the 15 frameworks, all but two^36,38^ used categories and hierarchies to organise their frameworks. The fewest categories were three and the most were 12. Many of the frameworks included similar categories and themes, which informed the creation of our framework. The most common category focused on financial elements (included in all except the Australian framework).^39^ The next most common category was scope (included in 13 frameworks), which set out what was included in disclosures (eg, are consultant lobbyists included in the definition, or is there a spending threshold to qualify as a lobbyist). Nine frameworks included elements of open data (ie, data accessibility).^20,31-35,39-41^ For some, this was limited to whether information was available online,^34^ whereas others had more detailed questions about how searchable and user-friendly the registers were.^35^

 In addition to indicators focusing on disclosure, several frameworks included other aspects of lobbying transparency, with 11 frameworks addressing enforcement and accountability^20,28-34,37,40,41^ and six included provisions around integrity and codes of conduct for lobbyists.^20,32,34,38,40,41^

**Figure 3:**
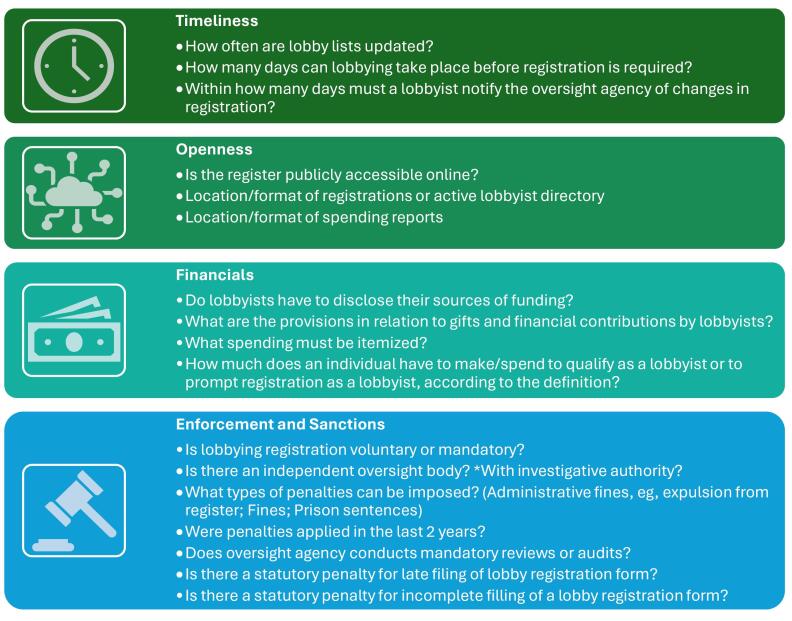
Questions Given the Most Weight in Lobbying Transparency Frameworks.

 Only three frameworks weighted indicators.^31,33,34^ The questions that were weighted the highest focused on timeliness of reporting, whether and how information is made available online, financial elements and enforcement and sanctions (See Figure 3). Of these, enforcement and sanctions were not included in our framework as it was outside the scope of our focus on disclosures.

###  Framework fOr Comprehensive and Accessible Lobbying 

 Our FOCAL was synthesised from the above 15 frameworks. It comprises eight categories and 50 indicators (Table 3). Each category corresponds to an aspect of transparent lobbying disclosures, which we elaborate on below. The first two categories (definitions and timeliness) can be assessed by viewing the reporting requirements for a register (eg, the legislation underpinning it), while the other categories can be assessed by viewing the register itself. In a complementary study we are testing the feasibility of applying FOCAL to evaluate government lobbying disclosures. While there are different ways that the indicators and categories could be grouped, our approach balances the conceptual similarities of the indicators in the categories with the practical aspect of where information is located in a register (to make application of FOCAL more straightforward).

**Table 3:** Framework fOr Comprehensive and Accessible Lobbying

**Categories**	**Indicators**	
*Scope*: The scope of what is included and excluded from the register	The following types of lobbyists are included in the register: professional lobbyists/consultants, in-house company lobbyists, in-house organisation lobbyists, professional consultancies, law firms, think tanks, research institutions, public entities, government agencies/employees	
	There is no (or low) financial or time threshold to qualify/exempt lobbyists from registration	
	The following are included as targets of lobbying: legislative branches, executive branch officials, Ministers, Deputy Ministers, members of parliament, Director-Generals and senior officials, staff, administrative branch/bureaucracy	
	A wide breadth of activities are included, eg, oral, written, electronic, virtual communications; organising meetings for others; events; phone calls and emails	
*Timeliness*: The frequency of lobbying disclosures	Changes (eg, registering/deregistering lobbyists, new clients) are updated close to real time (eg, daily)	
	Lobbying activities are disclosed close to real time (eg, daily)	
	Ministerial diaries are disclosed monthly (or more frequently)	
*Openness*: How easy it is to find and use information in the register	Lobbyist register is online	
	Diaries available online (eg, lobbyists, ministers, ministerial staff)	
	Available without registration, free to access, open license (eg, no limits to reuse), non-proprietary format (eg, CSV, not Excel), machine readable	
	Downloadable (eg, as files, database)	
	Searchable, simultaneous sorting with multiple criteria	
	Unique identifiers (eg, for lobbyists, individuals, organisations)	
	Linked or interconnected data (to other datasets, eg, campaign financing)	
	Historical data in lobbyist register is archived and published; downloadable	
	Changes or updates documented with a flagging system	
*Descriptors*: Descriptions and identifying elements of the individuals and organisations involved in lobbying	Full names of lobbyists/organisations, (not abbreviations or ambiguous names)	
	Contact details provided (eg, Address, telephone and/or website)	
	Legal form (eg, public, private, not-for-profit, NGO, government)	
	Company registration number	
	Sector (eg, transport, energy), sub sector	
	Type of lobbyist contract (eg, salaried staff, contracted)	
*Revolving door*: The movement between public and private sector employment	List of all prior public offices that lobbyists have held, dates when left office	
	Database of public officials who are subject to a ban on lobbying (eg, cooling off period)	
*Relationships*: The connections between the different actors involved in or benefiting from lobbying	Client list (for all consultant lobbyists and firms)	
	Names of all sponsors or members (for associations and representative groups)	
	List of board seats held (eg, in associations, companies)	
	Direct business associations with public officials, candidates or members of their households	
*Financials:* The flow of money spent and earned through lobbying activities	For consultant lobbyists & lobby firms	Total lobbying income (for consultant lobbyists/lobby firms)
		Lobbying income per client
	Income sources (eg, including government agencies, grant-making foundations, companies) and amount received	
	Number of lobbyists employed/contracted (total and full-time equivalent)	
	Amount of time spent on lobbying	
	Total lobbying expenditure (both in-house and consulting)	
	Compensated/uncompensated lobbying activities	
	Expenditure per issue	
	Expenditure on membership/sponsorship of organisations that lobby (eg, trade associations)	
	Expenditures beneﬁtting public ofﬁcials or employees including financial/non-financial gifts and support, employer/principal on whose behalf expenses were made	
	Campaign/political contributions, including in-kind	
*Contact log*: The activities of lobbyists	Organisation/interest(s) represented (beneficiary)	
	Names of persons contacted and their position/role	
	Institution/department contacted	
	If a meeting, names of all attendees	
	Date	
	Form (eg, in person meeting, video conference, phone call)	
	Location	
	Any materials that were shared, excluding commercially sensitive materials (before, during and after the meeting)	
	Topics/issues discussed	
	Outcomes sought (eg, legislation/policies supported/opposed)	
	Targeted areas of public policy or legislation, including a list of official legislative references/bill numbers/measures etc	

Abbreviation: NGO, non-governmental organization.

*Scope:* This category refers to the boundary of lobbyist registers (or other disclosure mechanism) and who or what is included or excluded. While this is not technically an aspect of disclosure, it fundamentally underpins what is or is not captured in a lobbying disclosure and is included for that reason. Indicators here focus on what activities are understood as lobbying, what branches of the government are covered (eg, only lobbying the legislature or also the executive), and how a lobbyist is defined. We note that some of these may be context dependent, for instance the target of lobbying may vary depending on the form of government (eg, a Westminster vs Presidential system). Likewise, what constitutes a low financial threshold for lobbying will depend on the country context. Of the frameworks we analysed, 13 of the 15 included elements related to scope, with only two not focussing on this aspect.^36,40^

*Timeliness:* This captures the frequency of lobbying disclosures. This includes how often regular reports are filed and published (eg, quarterly spending reports) as well more ad hoc activities, such as reporting meetings or changes to the registers (eg, adding or removing commercial clients). We include here the frequency of ministerial diaries (or other government contact log) as these are a complimentary disclosure mechanism that can reveal lobbying activities and can also help to verify their accuracy and completeness. Our recommendation for real time disclosures is based on recommendations made in the International Standards for Lobbying Transparency, which state that “The frequency of activity reporting should be set with the aim of allowing for the meaningful analysis and intervention from other parties (minimum quarterly, ideally close to real-time).”^41^ The aspect of timeliness was included in eleven frameworks, often as an feature of openness.^20,28,31,33-36,38-41^ We have separated timeliness from openness, as timeliness (like scope) is better assessed by viewing lobbying regulations.

*Openness:* This is fundamentally about how easy it is to find and use information in the register. We observed that openness is a more recent feature in the frameworks and has become more detailed, in part a function of increasingly sophisticated websites and online user experience. Eleven frameworks included this element, albeit in varying levels of detail.^20,30-37,40,41^ The initial indicators ask whether registers and diaries are online, acknowledging that this is rare internationally. The next series of indicators consider barriers to accessing the data (eg, cost, license) and how easy it is to search and analyse the data (eg, whether the data is downloadable in a structured format like.csv or whether it can be searched and filtered online). Two elements consider the ability to link lobbying data to other sets, in particular through the use of unique IDs (eg, a numerical ID that differentiates lobbyists with the same name or matches companies that lobby under multiple names). The final element considers how easy it is to access historical data or monitor changes in the data.

*Descriptors:* This category includes the biographical or descriptive elements provided in the register for lobbyists, lobby firms, commercial organisations, government targets, or other individuals and organisations that are involved in lobbying. Ten frameworks included this element.^20,31-33,35-38,40,41^

*Revolving door:* This captures whether lobbyists have had prior experience in government, or whether government officials have come from the private sector. While similar to the relationships’ category, this particular type of relationship is often subject to specific regulations (eg, cooling off periods where former government officials are prohibited from working as lobbyists). The revolving door is also more closely related to issues of public integrity rather than the beneficiaries of lobbying. For these reasons, we made this a standalone category. Only four frameworks specified that information about the revolving door should be disclosed in lobbyist registers.^32,36,37,41^ In contrast, the Hired Guns framework^33^ asked whether there was a ‘cooling off’ period imposed, and the Laboutková and Vymětal framework^20^ had the most detailed section on revolving door provisions as part of their broader transparency framework, however they were not incorporated into the specific recommendations for the design of lobbyist registers.

*Relationships: *This category documents the range of interests involved in lobbying. This includes potential conflicts of interest based on the relationships between lobbyists and the targets of their lobbying. It also recognises that the ultimate beneficiaries of lobbying may not be directly involved in lobbying activities. For example, the clients of lobbying firms or the members of industry associations and peak bodies who lobby on their behalf (See Figure 2 in Methods). 11 frameworks included this aspect of disclosure.^20,31-37,39-41^

*Financials:* This corresponds to the flow of money spent and earned through lobbying activities. We note that indicators about the money earned through lobbying are applicable to consultant lobbyists and lobby firms who are paid to lobby. These indicators help to establish who spent money doing what activity for what purpose. Some questions also capture other lobbying costs and resources of an organisation, such as the number of lobbyists employed, and the hours spent lobbying. We note that this particular category is especially US-centric, with many indicators originating from the Hired Guns framework, designed to evaluate US state lobbying regulations.^33^ 14 frameworks included financial aspects, with only the recommendations made for the Australian state of Victoria omitting finance.^39^

*Contact log: *This is about the activities of lobbyists, including meetings (in-person and virtual), phone calls, emails, and other efforts to access and influence the government targets of lobbying. Of particular importance is the indicator about the purpose or desired outcomes of the contact. While no framework provided specific examples of how this should be done, several were quite explicit on this point, for instance the Alliance for Lobbying Transparency and Ethics Regulation (ALTER-EU) “Organisations should be required to provide precise information on the main legislative proposals they are lobbying on, including a list of official legislative references” and Holman “The specific content of communications with contacted officials or entities or a summary thereof.”^32,36^ 13 Frameworks included this aspect of disclosure, with only the Hired Guns and Bednářová frameworks omitting it.^30,32^

## Discussion

 There is a rich history of scholarly and NGO scrutiny and analysis of lobbying practices. Despite this scrutiny, we identified relatively few frameworks that evaluate lobbying disclosure and transparency or set out guidelines for what should be included in a lobbyist register (or other disclosure system). Perhaps this should not be surprising, given that many countries have only recently required the publication of lobbying activities, and most have no law requiring lobbying disclosures.^2,19^

 Most of the frameworks we identified, especially those from NGOs, focused on evaluating or reforming regulations, rather than analysis and improvement of the practicalities of disclosure. FOCAL (our framework) offers a complementary tool that helps to consider how lobbyist registers could be designed to provide relevant and detailed information that is easy to search and analyse. It also helps to strengthen the evidence base underpinning transparency regulations by offering detailed methods for our framework (something that was lacking in most of the frameworks we assessed).

 FOCAL seeks to strike a balance between fostering as much transparency as possible while also minimising the administrative and reporting burden (both for governments as well as lobby groups or advocates that might have fewer resources). On one hand, if individual citizens or small organisations are required to complete detailed reports about low levels of advocacy, this can create a barrier to democratic participation in government. On the other hand, not holding all individuals and organisations to the same standard, risks loopholes being exploited to hide lobbying activities. This can be seen in tobacco control, where the tobacco industry has been formally excluded from policy-making in many countries, so the industry began using a range of seemingly independent groups to lobby on their behalf.^42^ With few exceptions (such as advocates from vulnerable groups such as refugees or whistleblowers), transparency rules should be applied broadly.

 To date, much of the intelligence about lobbying strategies has come from internal industry documents (such as those housed in the University of San Fransisco library from the tobacco, opioid, fossil fuel and other industry sectors). From these, public health advocates have been able to understand the strategies used to access and influence policy-makers, shape policy agendas, and delay or defeat legislation that threatens their industry.^43^ Comprehensive and detailed lobbying disclosures could help reveal the similarities and differences in how diverse industry sectors engage in politics, which in turn can help public health advocates develop counter strategies to protect public health legislation from commercial interference.^44,45^

 Comprehensive disclosure requirements could prove to be a double-edged sword for public health advocates and others seeking better intelligence about commercial lobbying. While on the one hand advocates would be better informed, commercial actors would likewise have better intelligence about how and why public health advocates lobby governments.^46^ We will continue to explore the question of how best to balance the ideal of transparency with the practical reality of (sometimes) limited resources on the part of lobby groups and governments in the next stage of this project, where we apply FOCAL to analyse the lobbying disclosure practices of governments. This will allow us to test how easy FOCAL is to implement, an important measure of a framework’s reliability and reproducibility across contexts.^21^ It will also allow us to benchmark government practice and identify examples of best practice that other governments could emulate.

 While we have not added weights to our indicators, we propose that two categories are especially important for transparency lobbying. If governments have limited resources (such as many LMICs) to implement all aspects of FOCAL, we suggest they prioritise *scope* and *contact logs*. First, the scope of lobbying regulations fundamentally determine the potential breadth of information. For countries like the United Kingdom and Australia that limit the scope of lobbyists to “third party lobbyists” (ie, those employed by a professional lobby firm), this excludes a huge segment of the lobbying population that work directly for companies or associations (often called “in-house” lobbyists).^18^ The second category we prioritise is contact logs, as these provide (or should provide) a record of which government officials are contacted, whose interests are represented by the lobbyist, and the purpose of the meeting. Based on a preliminary analysis of governments requiring lobbying contact logs, we suggest that Chile is an exemplary model.^46^ A contact log can also provide information covered elsewhere in FOCAL. Several descriptors (eg, names, position) are included in contact logs. Likewise, information about relationships, in particular the ultimate beneficiary of lobbying (See Figure 2) should be also included in a well-designed contact log. This could go a long way towards preventing so-called dark lobbying, where lobby groups do not disclose their clients or associations camouflage their sponsors and clients (a well-known strategy of the tobacco industry).^47,48^

 This first iteration of FOCAL is conceptual – the next logical step is to apply the framework to assess government lobbying disclosures in practice (this is the next phase of our research project). Policy-makers can also use this framework to assess their own lobbying regulation (if it exists) and what aspects are missing or require strengthening. Many countries lack lobbying registers, and in those cases FOCAL offers a template for what could be developed to improve lobbying transparency.

 In the absence of robust lobbying transparency regulations in most countries, there is an opportunity for researchers and NGOs to step in and fill the gap. Prominent examples include organisations like OpenSecrets and Transparency International, which have developed websites to link and display lobbying data.^49,50^ In the academic space, a data science team at the Massachusetts Institute of Technology developed the interactive website LobbyView.^51^

 One limitation of FOCAL is that it is unweighted (ie, all indicators are equally important). Only a few of the frameworks we analysed had weighted indicators, suggesting that a useful area for future research is to assign values to the indicators to highlight those that are the highest priority, such as through a Delphi study or other methods to reach consensus. We will return to this question around weighting indicators in the second phase of this project where we will be able to assess how governments disclose lobbying activities in practice and which indicators have the strongest and weakest implementation. Likewise, some aspects of FOCAL are more subjective than others. For instance, what is a “low” financial or time threshold to qualify/exempt lobbyists from registration? This is a question we will consider in the next stage of the project when we implement FOCAL and benchmark government disclosure practices.

 A further limitation is that FOCAL focuses on improving one aspect of lobbying disclosure and transparency: assessing *what* information about lobbying is disclosed and *how* it is publicly shared. Yet as we found in our scoping review, many other elements are crucial to foster transparent lobbying. Alongside comprehensive, timely and accessible information about lobbying, we also require enforcement mechanisms, ethical codes of practice and complementary transparency rules, such as whistleblower protections.^20,52^ Indeed, the finding that enforcement and sanctions were heavily weighted (in the three frameworks that ranked their indicators) emphasises the need for legal instruments with mandatory requirements and penalties to ensure compliance with disclosure requirements. Further, while transparency is important, it is not a panacea, with Hood et al^53^ observing that transparency can be thought of as a tool for achieving goals, rather than a goal in itself. To our knowledge, there are no studies analysing the impact of lobbying regulations on the behaviours of government officials. If more transparency is required, does this shift norms and behaviours in terms of whether and how they engage with commercial or other lobbyists? We suggest this would be an interesting area for future research to assess the impact of transparency regulations on the practice of lobbying.

## Conclusions

 This paper is the first to comprehensively identify and analyse the range of scholarly and NGO-led frameworks to assess lobbying transparency and disclosure. Inspired by our own challenges accessing and analysing information about commercial lobbying, we develop a novel framework, FOCAL, that sets out the key elements that governments should be disclose about lobbying to ensure that relevant information is accessible and user-friendly. We hope that FOCAL provides a resource for policy-makers and advocates seeking to strengthen transparency measures.Comprehensive criteria for lobbying disclosures provide a guide for research and advocacy efforts to evaluate and/or reform government transparency regulations.

 We recognise that lobbying disclosures and transparency more generally are only part of a holistic strategy to improve public integrity and reduce the risk of policy capture. Important also are measures to foster more inclusive and equitable opportunities for the public to engage in policy-making, ie, making government more representative and participatory.^52^ Nonetheless, transparency is an important first step towards reducing public sector corruption and ensuring government actions are in the public interest.^54^

## Ethical issues

 Not applicable.

## Conflicts of interest

 JLN is the recipient of a fellowship from the Victorian Health Promotion Foundation.

## Supplementary files

Supplementary file 1. Database and Grey Literature Search Strategies.
