# Supplementary material for: Lobbying in the Sunlight: A Scoping Review of Frameworks to Measure the Accessibility of Lobbying Disclosures
Source: Int J Health Policy Manag. 2024 Oct 8;13:8497. doi: 10.34172/ijhpm.8497 (PMC11549566; doi:10.34172/ijhpm.8497)
Supplement: Supplementary file 1 — Database and Grey Literature Search Strategies. [file ijhpm-13-8497-s001.pdf]

Article title: Lobbying in the Sunlight: A Scoping Review of Frameworks to Measure the Accessibility of Lobbying Disclosures

Journal name: International Journal of Health Policy and Management (IJHPM)

Authors' information: Jennifer Lacy-Nichols[1]*, Hedeeyeh Baradar[1], Eric Crosbie[2], Katherine Cullerton[3]

[1]Centre for Health Policy, Melbourne School of Population and Global Health, The University of Melbourne, Melbourne, VIC, Australia.

[2]School of Public Health, University of Nevada Reno, Reno, NV, USA.

[3]School of Public Health, The University of Queensland, Brisbane, QLD, Australia.

***Correspondence to:** Jennifer Lacy-Nichols; Email: jlacy@unimelb.edu.au

Citation: Lacy-Nichols J, Baradar H, Crosbie E, Cullerton K. Lobbying in the sunlight: a scoping review of frameworks to measure the accessibility of lobbying disclosures. Int J Health Policy Manag. 2024;13:8497. doi:10.34172/ijhpm.8497

Supplementary file 1. Database and Grey Literature Search Strategies

Category groups (and synonyms):

#	Concept	Search terms
1	Framework	Framework* OR Model* OR Principle* OR Index OR Assessment* OR Evaluation* OR Schem* OR Criteri* OR Indicator* OR Indice*
2	Lobbying	Lobby*
3	Transparency	Transparen* OR Disclos* OR Register* OR Registr* OR “open agenda*” OR “open diar*” OR “contact log*” OR Log OR Agenda* OR Diar* OR Contact* OR "lobby* regulation*" OR "lobby* code*"

Note: we originally searched a wider range of terms associated with lobbying (“interest group*” OR “pressure group*” OR “outside group*” OR advoc* OR “revolving door” OR “corporate influence”), however, these returned irrelevant results that analysed the practice of lobbying, rather than government transparency and disclosure mechanisms. Separate searches of these terms combined with “open agenda” and other transparency terms yielded no relevant results.

Database search strategies

Search terms	Platform or URL	Date	Results
(TITLE-ABS-KEY (framework* OR model* OR principle* OR schem* OR criteri* OR indicator* OR indice* OR index OR assessment* OR evaluation* OR structure*) AND TITLE-ABS-KEY (lobby*) AND TITLE-ABS-KEY (transparen* OR disclos* OR register* OR registr* OR log OR agenda* OR diar* OR contact*) OR ALL ("lobbyist code" OR "Lobbying code" OR "contact log" OR "open agenda" OR "open diary" OR "lobbying regulation" OR "Lobbyist regulation"))	Scopus	8/03/2023	660
(((TS=(Framework* OR Model* OR Principle* OR Schem* OR Criteri* OR Indicator* OR Indice* OR Index OR Assessment* OR Evaluation*)) AND TS=(Lobby*)) AND TS=(transparen* OR disclos* OR register* OR registr* OR log OR agenda* OR diar* OR contact*) OR ALL=("lobbyist code" OR "Lobbying code" OR "contact log" OR "open agenda" OR "open diary" OR "lobbying regulation" OR "Lobbyist regulation"))	Web of Science	8/03/2023	538
abstract(Framework* OR Model* OR Principle* OR Schem* OR Criteri* OR Indicator* OR Indice* OR Index OR Assessment* OR Evaluation*) AND abstract(lobby*) AND abstract(transparen* OR disclos* OR register* OR registr* OR log OR agenda* OR diar* OR contact*) OR ("lobbyist code" OR "Lobbying code" OR "contact log" OR "open agenda" OR "open diary" OR "lobbying regulation" OR "Lobbyist regulation")	ProQuest	8/03/2023	1088
(Abstract: Framework* OR Model* OR Principle* OR Index OR Assessment*) AND (Abstract: lobby*) AND (Abstract: transparen* OR disclos* OR register* OR registr* OR log OR agenda* OR diar* OR contact*)	JSTOR	8/03/2023	53
(All fields: Framework* OR Model* OR Principle* OR Index OR Assessment*) AND (Abstract: lobby*) AND (All fields: "lobbyist code" OR "Lobbying code" OR "contact log" OR "open agenda" OR "open diary" OR "lobbying regulation" OR "Lobbyist regulation")	JSTOR	8/03/2023	39
(AB (framework* OR model* OR principle* OR schem* OR criteri* OR indicator* OR indice* OR index OR assessment* OR evaluation* OR structure*) AND AB (lobby*) AND AB (transparen* OR disclos* OR register* OR registr* OR log OR agenda* OR diar* OR contact*) OR ("lobbyist code" OR "Lobbying code" OR "contact log" OR "open agenda" OR "open diary" OR "lobbying regulation" OR "Lobbyist regulation"))	Business Source Complete	8/03/2023	156

*Where possible we limited our database searches to scholarly journals to ensure a manageable scope of analysis.

Grey literature searches

Google Advanced Search

Note: filter was applied to limit to pdf documents (to restrict to reports)

Searches "All results" – first 10 pages, representing 100 results screened

#	Search	Date	# results	# results screened	# new potentially relevant records
1	(Framework OR Model OR Principle OR Index) AND Lobby* AND (transparency OR disclosure OR register)	28/02/2023	About 61,800,000 results	100	16
2	(Framework OR Model OR Principle OR Index) AND Lobby* AND ("open agenda" OR "open diary" OR "contact log" OR "lobbying code" OR lobbyist code")	28/02/2023	About 1,260,000 results	100	9

Targeted website searches

Each website was searched for the terms transparency and lobby. In some cases these terms were searched together, whereas in others the search interface required separate searches. Where separate searches were conducted these are documented in separate rows in the below table.

Organisation	Website	Date	Search	# results	# results screened	# new potentially relevant records
OECD	https://www.oecd.org/australia/	6/4/2023	Search: lobby transparency	4,680	100	32
IDEA	https://www.idea.int/	6/4/2023	Search: lobby transparency	4	4	1
The Center for Public Integrity	https://publicintegrity.org	18/04/2023	Search: transparency	822	100	1
Centre for Public Integrity	https://publicintegrity.org.au/	6/4/2023	Research: Money in politics; National Integrity Commission; Accountability Institutions; Executive Power	40	40	17
Australian Democracy Network	https://australiandemocracy.org.au/	6/4/2023	Searched 'resources'	9	9	6
Alliance for Lobbying Transparency and Ethics Regulation (ALTER-EU)	https://www.alter-eu.org/	6/4/2023	Search: lobby transparency	270	100	26
Centre for Research on Multinational Corporations	https://www.somo.nl/	6/4/2023	Search: lobby transparency	27	27	4
Open Secrets	https://www.opensecrets.org/	5/4/2023	Search: lobby transparency	4,420	100	7

CorpWatch	https://www.corpwatch.org/	5/4/2023	Search: lobby transparency	25	25	2
Corporate Accountability	https://corporateaccountability.org/	5/4/2023	Search: lobby transparency	3	3	0
Corporate Europe Observatory (CEO)	https://corporateeurope.org/en	31/3/2023	Search: lobby transparency	600	100	23
InfluenceMap	https://influencemap.org/	31/3/2023	Search: lobby transparency	2	2	0
Friends of the Earth	https://www.foe.org.au/?gclid=CjwKCAjw5pShBhB_EiwAvmnNV3QxKspWNkSyxpjY_57eQfwng1wGmhVpRldLdR7U9IevpRS1UiF_VhoCOLkQAvD_BwE	31/3/2023	Search: lobby transparency	26	10	0
Multinationals Observatory	https://multinationales.org/en/	31/3/2023	Search: lobby transparency	9	9	2
Transnational Institute	https://www.tni.org/en	31/3/2023	Search: transparency (limit to publication)	3	3	1
Transparency International	https://www.transparency.org	31/3/2023	Advanced search (limit to publication): lobby	11	11	9
Sunlight Foundation	https://sunlightfoundation.com/	31/3/2023	Search: lobby, transparency	0	0	0
International standards for lobbying regulation	https://lobbyingtransparency.net/	31/3/2023	No search, drop down to print pdf	1	1	0
Open Knowledge Foundation	https://au.okfn.org/	31/3/2023	Search: lobby, transparency, lobby transparency	0	0	0
Access Info	https://www.access-info.org/page/22/?s=lobby+transparency&lang=en	30/3/2023	Search: lobby transparency	218	100	26
US Right to Know	https://usrtk.org/	30/03/2023	Search: lobby transparency	128	100	0
Public Citizen	https://www.citizen.org/	30/3/2023	Search: lobby transparency (limit to narrow results of reports)	5	5	2
